# Deregulated Expression of Aurora Kinases Is Not a Prognostic Biomarker in Papillary Thyroid Cancer Patients

**DOI:** 10.1371/journal.pone.0121514

**Published:** 2015-03-25

**Authors:** Enke Baldini, Chiara Tuccilli, Natalie Prinzi, Salvatore Sorrenti, Laura Falvo, Corrado De Vito, Antonio Catania, Francesco Tartaglia, Renzo Mocini, Carmela Coccaro, Stefania Alessandrini, Susi Barollo, Caterina Mian, Alessandro Antonelli, Enrico De Antoni, Massimino D’Armiento, Salvatore Ulisse

**Affiliations:** 1 Department of Experimental Medicine, “Sapienza” University of Rome, Rome, Italy; 2 Department of Surgical Sciences, “Sapienza” University of Rome, Rome, Italy; 3 Department of Public Health and Infectious Diseases, “Sapienza” University of Rome, Rome, Italy; 4 Department of Medicine, University of Padua, Padua, Italy; 5 Department of Clinical and Experimental Medicine, University of Pisa, Pisa, Italy; University of L'Aquila, ITALY

## Abstract

A number of reports indicated that Aurora-A or Aurora-B overexpression represented a negative prognostic factor in several human malignancies. In thyroid cancer tissues a deregulated expression of Aurora kinases has been also demonstrated, butno information regarding its possible prognostic role in differentiated thyroid cancer is available. Here, weevaluated Aurora-A and Aurora-B mRNA expression and its prognostic relevance in a series of 87 papillary thyroid cancers (PTC), with a median follow-up of 63 months. The analysis of Aurora-A and Aurora-B mRNA levels in PTC tissues, compared to normal matched tissues, revealed that their expression was either up- or down-regulatedin the majority of cancer tissues. In particular, Aurora-A and Aurora-B mRNA levels were altered, respectively, in 55 (63.2%) and 79 (90.8%) out of the 87 PTC analyzed.A significant positive correlation between Aurora-A and Aurora-B mRNAswas observed (p=0.001). The expression of both Aurora genes was not affected by the BRAF^V600E^ mutation. Univariate, multivariate and Kaplan-Mayer analyses documented the lack of association between Aurora-A or Aurora-B expression and clinicopathological parameterssuch as gender, age, tumor size, histology, TNM stage, lymph node metastasis and BRAF status as well asdisease recurrences or disease-free interval. Only Aurora-B mRNA was significantly higher in T(3-4) tissues, with respect to T(1-2) PTC tissues. The data reported here demonstrate that the expression of Aurora kinases is deregulated in the majority of PTC tissues, likely contributing to PTC progression. However, differently from other human solid cancers, detection of Aurora-A or Aurora-B mRNAs is not a prognostic biomarker inPTC patients.

## Introduction

The incidence of differentiated thyroid cancers (DTC) has been increasing over the last decades, mainly due to the increasing ability to diagnose malignant transformation in small non-palpable nodules [1–4]. DTC comprise two main histological entities, the rare follicular thyroid carcinoma (FTC) and themore common papillary thyroid carcinoma (PTC). Following dedifferentiation DTCare assumed to generate poorly DTC (PDTC) and highly aggressive anaplastic thyroid carcinomas (ATC) [5–6]. Relevant molecular alterations encountered in thyroid cancer progression comprise gene rearrangements of tyrosine kinase receptors, such as the RET/PTC and NTRK1, activating point mutations of the RAS and BRAF genes, and the oncogenic fusion protein PAX8-PPARγ [7].

The prognosis of DTC patients is usually favorable, with a 10-year-survival rate for approximately 90% of them. Nonetheless, about 20% of patients face disease recurrence and DTC-related deaths [8]. The stratification and prognosis of DTC patients rely on clinicopathological variables such as the patient’s age, tumor size, histology, lymph nodal or distant metastasis [8–11]. These parameters, however, are capable of providing only a rough prediction of the disease outcome, placing patients with very different disease-specific progression and survival times within the same risk group. Similarly, they fail to predict the risk of cancer recurrence. Therefore, the identification of new prognostic molecular biomarkers able to testify tumor aggressiveness is required [11–17].

The genetic instability leading to cell aneuploidy and transition to more aggressive phenotypes represents a hallmark of solid cancers including thyroid carcinomas [18–22].In fact, the number and the frequency of chromosomal abnormalities observed in thyroid cancers increase from DTC to PDTC and ATC [5, 20]. Different mitotic kinases, whose expression or function has been found altered in cancer cells, are held responsible for tumor genetic instability. These include the three Aurora kinase family members, Aurora-A, -B and -C, implicated in the regulation of multiple aspects of chromosome segregation and cytokinesis [23]. During the cell cycle, their expression is closely regulated, being maximal in the G2/M phase, while their rapid degradation at the end of mitosis by the ubiquitin-proteasome pathway is required to permit the cell to enter into a new cell cycle [23]. Aurora-A localizes onto the duplicated centrosomes and is involved in mitotic entry, centrosome and spindle maturation, while Aurora-B associates with chromatin where it forms the so-called chromosomal passenger complex (CPC) with other proteins such as INCENP, survivin and borealin, participating in chromosome condensation [23]. Moreover, during the transition from anaphase to telophase, Aurora-B plays a role in mitotic spindle dynamics, connections of chromosomes to spindle microtubules, and cleavage furrow. Aurora-C is expressed mainly in testis and, similarly to Aurora-B, it has been shown to join the CPC in mitotic cells [23]. Given the crucial tasks of Aurora kinases in all mitotic stages, their dysfunction and/or dysregulation are held responsible, at least in part, for the abnormal cell divisions and aneuploidy observed in malignant cells. In agreement with this, overexpression of Aurora-A and/or Aurora-B has been shown to associate with a poor prognosis in several human malignancies, including breast, gastric, prostate, head and neck, bladder, ovarian, colon, adrenocortical and lung cancers [24–31].

Data from our own and other research groups previously showed an altered expression of Aurora kinases in different thyroid cancer derived cell lines and tissues [32–35]. Although these kinases are now emerging as promising new therapeutic targets for thyroid cancer treatment, no attempt has been madeso far to evaluate the possible prognostic value of Aurora kinases in PTC patients [23, 36–39]. In the present studywe evaluated, by means of quantitative RT-PCR, the expression level of Aurora-A and Aurora-B in a case study of 87 PTC tissues matched against normal tissues. Data were then correlated with the clinicopathological parameters and with the disease-free interval of patients. In addition, since the BRAF^V600E^ mutation, the most frequently encountered alteration in PTC, was recently shown to induce the expression of Aurora-B in melanoma cells, the effects of the BRAF mutation on the expression of Aurora kinases in PTC tissues was also evaluated [40].

## Materials and Methods

### Tissue samples, histology and patient’s staging

The case study included 87 consecutive patients; normal and matched tumor thyroid tissues were obtained from surgical specimens of 19 males and 68 females (age range 11–83 yrs, median 44.21yrs) who underwent total thyroidectomy for PTC in the Department of Surgical Sciences, “Sapienza” University of Rome (n = 31), or in the Department of Medical and Surgical Sciences, University of Padua, Italy (n = 56).All patients gave their written informed consent. For three underage patients the written informed consent was obtained from their parents. The study was approved by the Policlinico Umberto I hospital ethical committee (Ref. 2615). Tissue samples were collected, frozen in liquid nitrogen and stored at −80°C. Of the 87 PTC patients, 76 exhibited the classical, 10 the follicular, and 1 the tall cell variant. Two different histopathologists made the histological diagnoses independently according to the World Health Organization classification and in blind manner with respect to Aurora kinase expression [41]. At the time of surgery, lymph node metastases were found in 38 patients. Following TNM staging, 54 patients were identified as being at stage I, 1 at stage II, 25 at stage III and 7 at stage IV. Forty to fifty days after surgery, all patients underwent radioiodine therapy and the subsequent whole body scan (WBS) showed the absence of metastases. Patients then started thyroid hormone replacement therapy. To ascertain their disease-free condition, 4 to 5 months later all the patients underwent neck ultrasound and serum thyroglobulin (Tg) measurement. Recurrences were diagnosed by fine-needle aspiration (FNA) cytology, ^131^I WBS or histological analysis following surgical resection of the lesion. Of the 87 patients, the follow-up (median 63months, range 8-133months) was available for 78 (18 males and 60 females with a median age of 44yr), 52of whom were at TNM stage I-II and the remaining 26 at stage III-IV. The lower limit of times-to-recurrence started at 6 months. During the follow-up 21 recurrences were recorded, 17 being cervical lymph nodes, diagnosed by FNA cytology, and 4 lung metastases, diagnosed by WBS.

### Determination of BRAF^V600E^ mutation

Genomic DNA was extracted from the frozen tissues using the DNeasy Blood and Tissues kit according to the manufacturer’s protocol. The BRAF status of exon 15 was assessed by both direct sequencing and mutant allele-specific PCR amplification for the T to A substitution at nucleotide 1799 (V600E), using the procedure previously describedand in blind manner with respect to Aurora kinase expression[42].

### PCCL3 cell culture

The well-differentiated, non-transformed rat thyroid epithelial cell line PCCL3 was a gift from Dr. J.A. Fagin (Memorial Sloan-Kettering Cancer Center, New York). The cells were propagated in H4 complete medium consisting of Coon’s medium/F12 high zinc supplemented with 5% fetal bovine serum, 0.3 mg/ml L-glutamine, 1 mIU/ml TSH, 10 μg/ml insulin, 5 μg/ml apo-transferrin, 10 nM hydrocortisone, and penicillin/streptomycin. These cells conditionally express BRAF^V600E^ in a doxycycline-dependent manner [43]. The BRAF^V600E^ induction was obtained by adding to H4 complete medium doxycycline 1 μg/ml, and incubating the cells for 48 h.

### Extraction and analysis of mRNA

The thyroid tissues were homogenized with the ultra-turrax, and total RNA was extracted from the tissues or PCCL3 cells applying the acid guanidinium thiocyanate—phenol—chloroform method [44]. The first cDNA strand was synthesized from 5 μg of RNA with M-MLV reverse transcriptase and anchored oligo(dT)23 primers (Sigma Chemicals Co.). Parallel controls for DNA contamination were carried out omitting the reverse transcriptase. The templates obtained were used for quantitative PCR amplifications of the Aurora kinases and housekeeping genes employing the LightCycler instrument (Roche Diagnostics, Mannheim, Germany), the SYBR Premix Ex Taq II (TliRNase H Plus) (Takara, Otsu, Shiga, Japan) and specific primers listed in Table 1. Amplicon specificities were checked by automated DNA sequencing (Primm, San Raffaele Biomedical Science Park, Milano, Italy), evaluation of melting temperatures, and/or electrophoresis on 2% agarose gel containing ethidium bromide. Standard curves for all genes were achieved with five-fold dilutions of PCCL3 cells or mixed human thyroid tissue cDNA. Calculation of data for human thyroid tissues was performed by the Relative Expression Software Tool (REST 2009) using a normalization factor (NF) computed as the geometric media of 3 reference genes (GAPDH, RPL13A and SDHA), as previously described [45, 46]. The fold change of Aurora kinase expression for each tumor sample was referred to its normal counterpart. This analysis was performedin blind manner with respect to histological and clinical data of the patients. Fold variations between 0.8 and 1.2 were considered unchanged. Calculations of the data for the experiments on PCCL3 cells were carried out using the LightCycler relative quantification software 1.0 (Roche Diagnostics), and the fold expression of Aurora kinases for doxycycline-treated PCCL3 cells was normalized against non-treated cells. All the results are reported as mean±SEM and median values.

**Table 1:** Sequences, genomic positions, and amplicon sizes of the primers used in qRT-PCR for the target and reference genes.

Gene	Primers	Exon	Size (bp)
Human Aurora-A	Forward 5′-TTGGAAGACTTGGGTCCTTG-3′ Reverse 5′-TGGAGCTGTAGCCTTAACAGG-3′	1 2–3	211
Human Aurora-B	Forward 5′-AAAGAGCCTGTCACCCCATC-3′ Reverse 5′-CGCCCAATCTCAAAGTCATC-3′	3 5	155
Human GAPDH	Forward 5’-ATCATCAGCAATGCCTCCTG-3’ Reverse 5’-GGCCATCCACAGTCTTCTG-3’	6–7 8	136
Human RPL13A	Forward 5’-ACCGTGCGAGGTATGCTG-3’ Reverse 5’-TAGGCTTCAGACGCACGAC-3’	4–5 6	148
Human SDHA	Forward 5’-GCATAAGAACATCGGAACTGC-3’ Reverse 5’-GGTCGAACGTCTTCAGGTG-3’	12 13	147
Rat Aurora-A	Forward 5′-TGCTGCTTGGCTCAAATG-3′ Reverse 5′-TCCGACCTTCAATCATCTCC-3′	10 11	105
Rat Aurora-B	Forward 5′-ACATAAAGCCCGAGAACCTG-3′ Reverse 5′-ATCCGCCCTTCAATCATCTC-3′	2 3	145
Rat GAPDH	Forward 5′-AACCCATCACCATCTTCCAG-3′ Reverse 5′-GGAGATGATGACCCTTTTGG-3′	4 5	147

GAPDH, glyceraldehyde-3-phosphate dehydrogenase; RPL13A, ribosomalprotein L13a; SDHA, succinatedehydrogenasecomplex, subunit A.

### Statistical analysis

The non-parametric Mann Whitney test was used to calculate the statistical significance of differences in the expression levels of Aurora kinases in PTC with deregulated expression versus PTC with unchanged mRNA levels, and in wild type versus mutated BRAF samples. In addition, the association of the expression of Aurora kinases with gender, histology, lymph node metastasis, TNM stage or recurrences was evaluated by the Mann Whitney test. The analyses of the correlation between Aurora-A and Aurora-B mRNAs levels, as well as between these and age were performed using the Rho Spearman test. To assess the independent association of Aurora kinases with DFI, the Cox regression text was applied. The impact of Aurora kinase expression on the disease-free interval (DFI) was assessed by means of Kaplan-Meier analysis combined with Mantel-Cox log-rank. For the Kaplan-Meier analysis, Aurora kinase values were divided into three groups based on increased, unchanged or decreased kinase expression. All statistical analyses were carried out using the version 8.0 of the Stata software (Stata Corporation 2003, College Station, Tx). Results were considered significantly different if p values were lower than 0.05.

## Results

### Expression of Aurora kinases in papillary thyroid cancer (PTC) tissues

**Fig 1:**
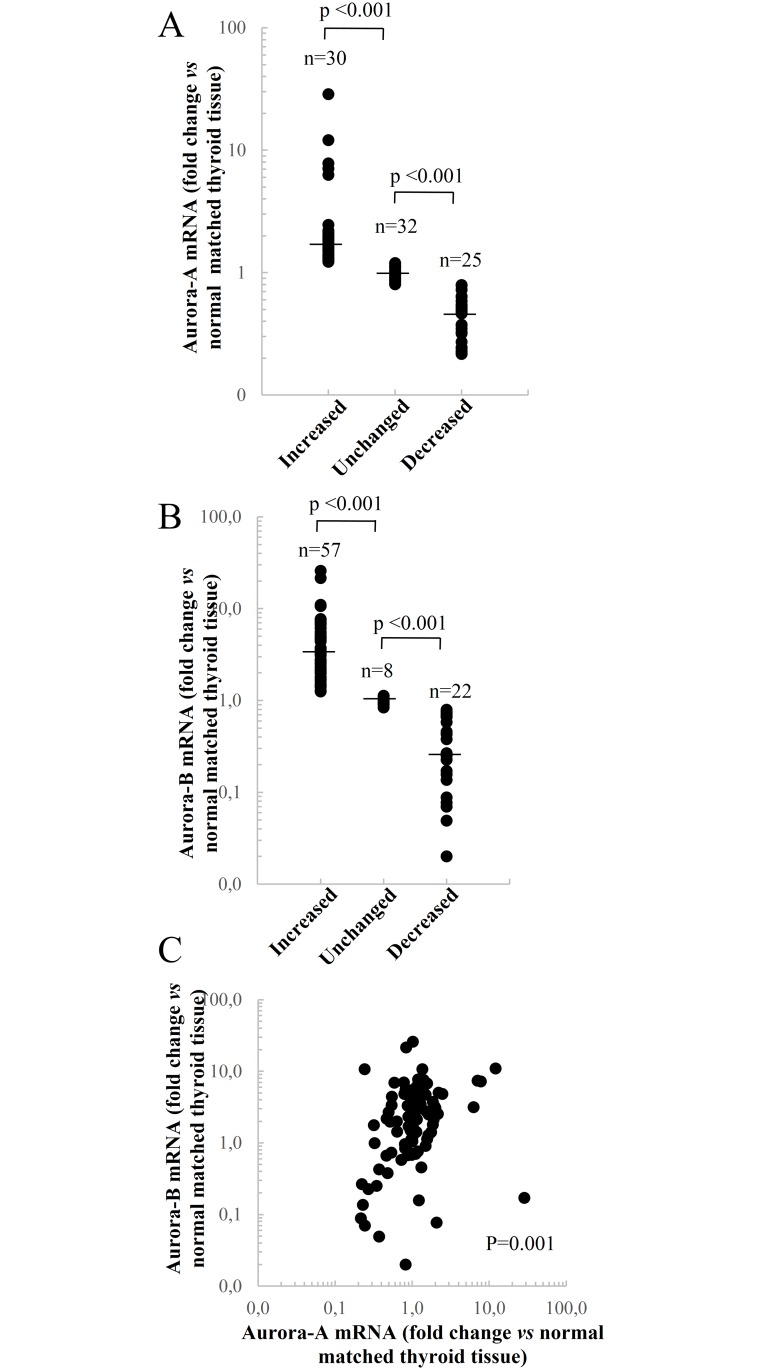
Expression of Aurora kinases in 87 papillary thyroid cancer tissues. A and B) Variations in the expression of Aurora-A and Aurora-B in papillary thyroid cancer tissues. The fold changes were calculated considering the Aurora-A or Aurora-B mRNAs levels observed in normal matched thyroid tissue equal to 1. The statistical evaluation of the data was performed usingthe non-parametric Mann-Whitney test. The small bars in the graph indicate the median values. C) Correlation analysis of Aurora-A and Aurora-B mRNAs in PTC tissues. The data were evaluated by applying the Rho Spearman test.

The analyses of Aurora-A and Aurora-B mRNA levels in PTC tissues, compared to their normal matched tissues,revealed that Aurora-A mRNA levelswere deregulated in 55 (63.2%) out of 87 PTC samples, with an increase in 30 and a decrease in 25 (Fig. 1A). Aurora-B mRNA levels were altered in 79 (90.8%) out of 87 samples, being up-regulated in 57 and down-regulated in 22 (Fig. 1B) of the cases. As reported in Fig. 1C, mRNA levels of Aurora-A and Aurora-B were positively correlated to each other (p = 0.001).

### BRAF^V600E^ mutation and Aurora kinase expression in PTC tissues

**Fig 2:**
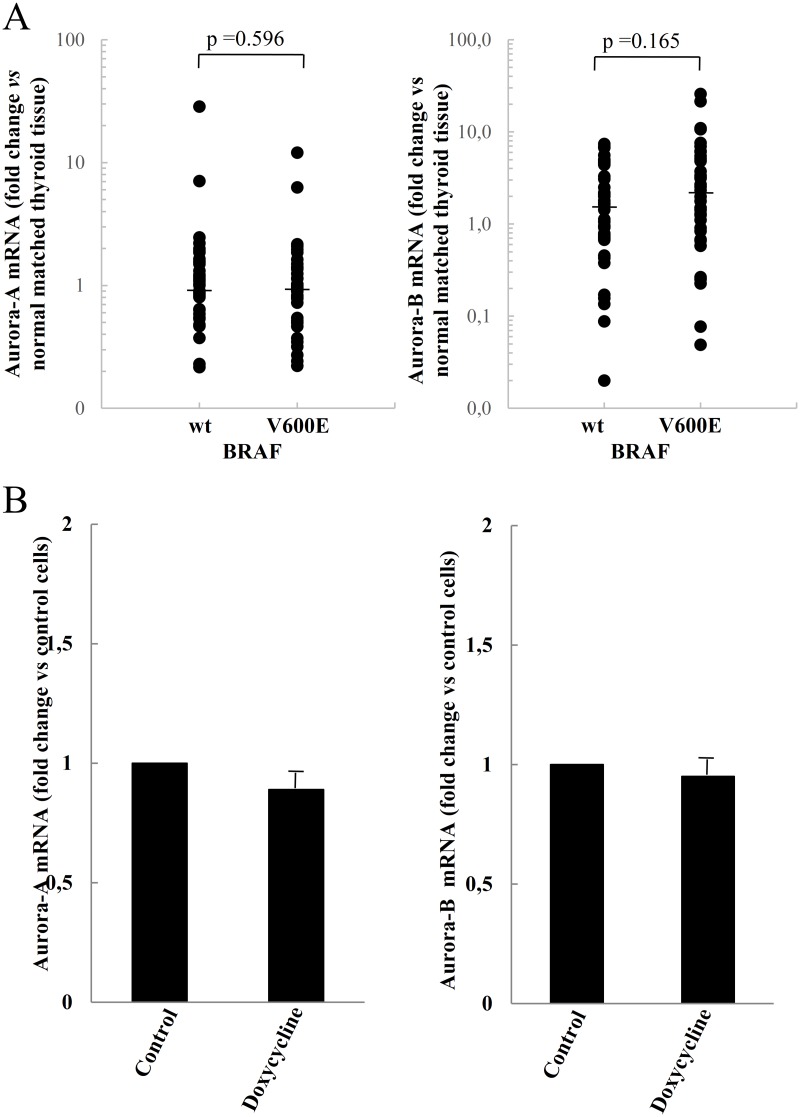
Lack of effects of BRAF^V600E^ on the expression of Aurora kinases. A) Expression of Aurora-A and Aurora-B in papillary thyroid cancer tissues with wild type (n = 38) or mutated BRAF (n = 37). The fold changes were calculated considering the Aurora-A or Aurora-B mRNAs level observed in normal matched thyroid tissue equal to 1. The statistical evaluation of the data was performed by applying the non-parametric Mann Withney test. The small bars in the graph indicate the median values. B) Effect of BRAF^V600E^ on Aurora kinases mRNAs in PCCL3 cells. The latter were induced to express BRAF^V600E^ in a doxycycline-dependent manner. The fold changes of Aurora kinase mRNA were normalized against the non-treated cells.

To assess the effect of BRAF^V600E^ mutation on the expression of Aurora-A and Aurora-B we first analyzed the mRNA level of both genes in BRAF^V600E^ PTC tumors (n = 37), compared with those harboring the wild type protein (n = 38) (Fig. 2A). The results showed that thepresence of the BRAF^V600E^mutation did not affect the expression levels of Aurora-A or Aurora-B in PTC tissues, compared with the wild-type BRAF PTC tissues. To corroborate these *in vivo* observations, we performed *in vitro* experiments on the well-differentiated, non-transformed rat epithelial cell line PCCL3, characterized by a doxycycline-dependent BRAF^V600E^ expression system [50]. In these cells the BRAF^V600E^ expression and subsequent induction of the MEK/ERK phosphorylation pathway appeared 12 h after the addition of doxycycline, and the total BRAF expression (endogenous wild-type + induced V600E mutant) at 48 h was estimated to be 2-fold greater than the control. As reported in Fig. 2B, the treatment of PCCL3 with doxycycline (1 μg/ml for 48 h) did not affect Aurora kinase mRNA levels.

### Prognostic relevance of Aurora kinase expression in PTC patients

**Fig 3:**
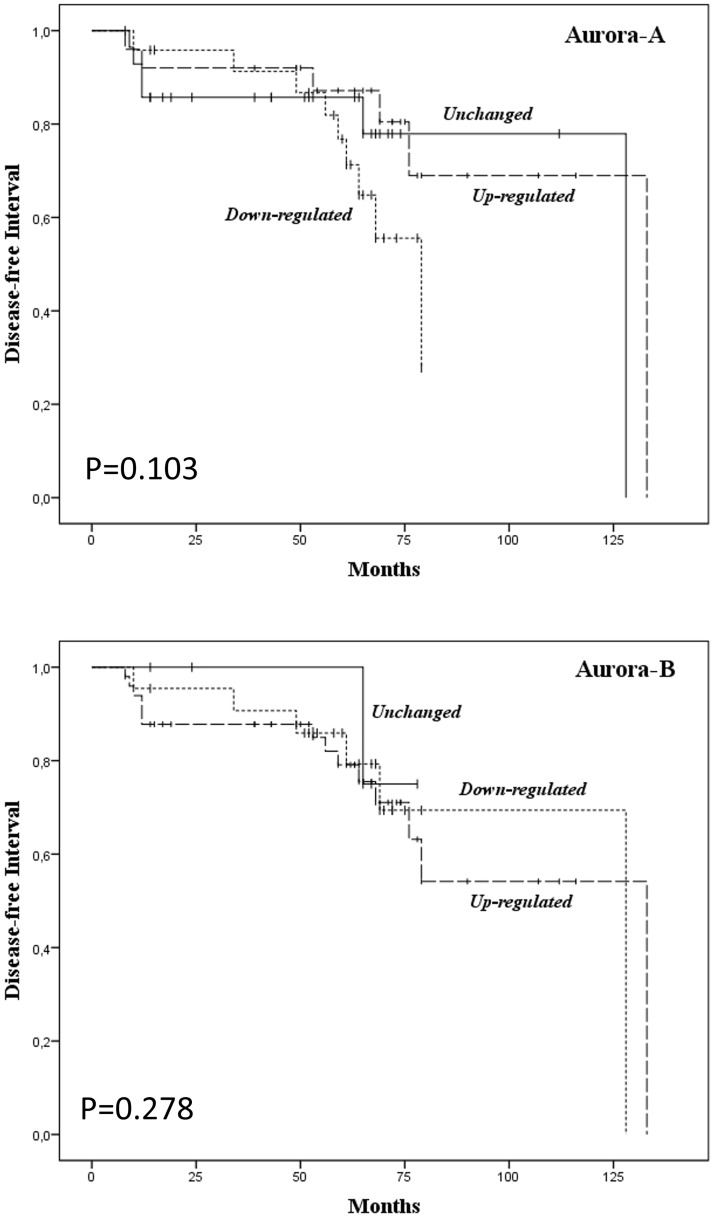
Aurora kinase mRNA levels and disease-free interval (DFI) in papillary thyroid cancer patients. Kaplan-Meier analysis combined with Mantel-Cox log-rank statistical test performed on 75 PTC patients followed-up from 8 to 133 months.

Variations in the expression of Aurora-A did not associate with any of the clinicopathological parameters analyzed (Table 2) while apositive correlation (p<0.001) was found between Aurora-B mRNA levels and tumor size (Table 2).The Kaplan-Meier analysis demonstrated no correlation between patients’ disease-free interval and Aurora kinase up- or down-regulation (Fig. 3). Multivariate analysis showed that increased or reduced expression of Aurora-A or Aurora-B, TNM stage, age or the BRAF^V600E^ mutation failed to predict disease outcome (Table 3). Only females showed a statistically significant reduction of the hazard ratio (HR 0.286) for disease recurrences (Table 3).

**Table 2:** Univariate statistical analysis of Aurora-A and Aurora-B expression and clinicopathological parameters in 87 PTC patients.

	Aurora-A	P value	Aurora-B	P value
**Gender**				
Male (n = 19)	1.93±0.62	0.463	4.33±1.35	0.423
Female (n = 68)	1.62±0.43		3.08±0.42	
**Age (years)**	Corr. Coeff. 0067	0.537	Corr. Coeff. 0.024	0.823
**Histology**				
Classic variant (n = 76)	1.68±0.39	0.285	3.35±0.47	0.946
Other variants (n = 11)	1.79±1.13		3.15±1.04	
**BRAF**				
Wild type (n = 38)	1.94±0.74	0.596	2.33±0.349	0.165
V600E (n = 37)	1.43±0.34		4.31±0.923	
**Tumor size**				
T(1–2)	1.80±0.79	0.366	1.63±0.24	<0.001
T(3–4)	1.60±0.29		4.51±0.68	
**Lymphnode metastasis**				
No (n = 49)	1.89±0.60	0.383	2.95±0.371	0.906
Yes (n = 38)	1.41±0.30		3.87±0.89	
**TNM Stage**				
I-II (n = 55)	1.29±0.24	0.200	2.89±0.39	0.271
III-IV (n = 32)	2.36±0.89		4.14±0.99	
**Recurrences**				
No (n = 57)	1.68±0.45	0.692	3.35±0.54	0.203
Yes (n = *21*)	1.70±0.76		3.14±0.75	

Corr. Coeff.: correlation coefficient.

**Table 3:** Cox regression analysis of different variables with recurrences in PTC patients.

Variable	Hazard Ratio	95% CI	*p* value
Aurora-A high	0.879	0.236–3.270	0.848
Aurora-A low	2.276	0.666–7.779	0.190
Aurora-B high	2.222	0.238–20.776	0.484
Aurora-B low	2.017	0.161–21.319	0.560
TNM stage (III-IV)	1.177	0.252–5.508	0.836
BRAF^V600E^	0.577	0.208–1.597	0.290
Gender (Female)	0.286	0.099–0.826	0.021
Age	0.985	0.935–1.038	0.572

### Discussion

In previous studies, our and other research groups demonstrated a deregulated expression of Aurora kinases in differentiated and anaplastic thyroid cancer tissues [23]. This was confirmed in the present study where Aurora-A and Aurora-B gene expression was found either up-regulated or down-regulated (63.2% and 90.8%, respectively) in the majority of the papillary thyroid cancer (PTC) tissues analyzed, compared to their normal matched thyroid tissues. It is worth mentioning that either the up-regulation or down-regulation of Aurora kinases may prove proliferatively advantageous to thyroid cancer cells. In fact, different reports demonstrated that the amount of Aurora-A proteinin the centrosome is critical for its replication and mitotic functions, and that either a lack or excess of Aurora-Acan lead to abnormal mitosis as well as to chromosome segregation and cytokinesis defects [47].

It has recently been shown that in melanoma cells the expression of Aurora-B and Wee1-like protein kinase are induced by the presence of BRAF^V600E^[40]. Since BRAF^V600E^ mutation is frequently encountered in PTC,we also sought to determine whether the mutation in thyroid cancer cells was associated to increased expression of Aurora kinases. In our series, the BRAF status was assessed in 75 PTC tissues and BRAF^V600E^ was found in 37 (49.3%)of them. However, no difference in Aurora-A or Aurora-B expression levelswas found between wild type and BRAF^V600E^ PTC tissues. These observations were confirmedby *in vitro* experiments on rat thyroid PCCL3 cells, expressing the BRAF^V600E^ in a doxycycline dependent manner [43]. In fact, the treatment of these cells with doxycycline did not affect the expression level of either Aurora-A or Aurora-B.

Over the last decade, a number of reports indicated that the overexpression of Aurora-A or Aurora-B represents a negative prognostic factor in several human malignancies, including breast, gastric, prostate, head and neck, bladder, ovarian, colon, adrenocortical and lung cancers[25–32]. Fewer studies, however, associated the overexpression of Aurora-A or Aurora-B with a favorable prognosis in colorectal, gastric and ovarian carcinomas [48–50]. To date, no information regarding the possible prognostic role of Aurora kinases in differentiated thyroid cancer has been reported. In this context, we here evaluated the prognostic relevance of Aurora-A and Aurora-B mRNA levels in a series of 78 PTC, with a median follow-up of 63 months. Univariate analyses documented the lack of association between Aurora-A expression and clinicopathological parameters,including age, gender, tumor size, lymph node metastasis, histology, TNM, BRAF status and recurrences. Similarly, Aurora-B expression did not correlate with any of these parameters with the exception of tumor size, in which mRNA levels were significantly higher in T(3–4) tissues, with respect to T(1–2) tissues. Kaplan-Meyer and multivariate analyses confirmed that deregulated expression of Aurora kinases is not a prognostic biomarker of papillary thyroid cancer patients. In multivariate analysis female sex showed a significant protective effect (Hazard Ratio 0.286, p = 0.021). The latter is in agreement with two recent studies performed on large case-series showing the protective role of the female gender on disease-specific survival[51, 52].It has to be mentioned that a limit of the present study is the relative low number of patients analyzed which provide a statistical power (1-β) of 0.63.

## Conclusions

In conclusion, although the data reported hereneed to be confirmed by means of larger case-studies, they demonstrated that the expression of Aurora kinases is deregulated in the majority of PTC tissues, and probably contributes to PTC progression and cancer cell aneuploidy. However, differently from other human solid cancers, Aurora-A or Aurora-B expression at the mRNA level is not a prognostic biomarker in the case of PTC patients.
